# Subclinical atherosclerosis in young persons horizontally infected with HIV-1 during infancy

**DOI:** 10.1186/1742-4690-9-S1-P132

**Published:** 2012-05-25

**Authors:** Augustin Cupsa, Florentina Dumitrescu, Dina Maria Cupsa, Andreea Cristina Stoian, L Giubelan, Irina Niculescu, Cristina Iocu

**Affiliations:** 1 Infectious Diseases at University of Medicine and Pharmacy From Craiova, Craiova, Romania

## Objectives

To evaluate subclinical atherosclerosis and to identify the cardiovascular risk (CVR) profile in young adults horizontally infected with HIV-1 during infancy.

## Methods

Retrospective randomized study carried out between 31.12.2009 and 30.06.2010 on 56 HIV infected persons(HIP) parenterally infected with HIV-1 during 1988-1990, following ART, under surveillance of HIV/AIDS Regional Center – Craiova. Variables followed: history and clinical data, traditional and additional CVR factors, metabolic, immunological and virusological parameters, inflammation markers (hs-CRP), ultrasound data regarding carotid intima-media thickness (IMT). Twenty-six HIV-seronegative young adults were assigned as control group (CG) for metabolic parameters, hs-CRP and IMT.

## Results

General characteristics of the group: average age = 20.82 ± 1.1 years, equal distribution by gender, 47 HIP (83.93%) classified as clinical and/or immunological AIDS, 26 HIP (46.43%) with CD4>500/mm^3^, 40 HIP (71.43%) with undetectable RNA-HIV when evaluated, average ART duration = 9.09 ± 3.2 years, average number of ART regimens = 3.2 ± 1.63, 40 HIP (71.43%) experienced to protease inhibitors (PIs). In HIP – hs-CRP = 2.17 mg/l, equivalent with a moderate CVR, statistically different compared with CG (p<0.0001); IMT = 0.76 ± 0.12 mm in HIP vs 0.6 ± 0.11 mm in CG. From the traditional CVR factors dyslipidemia levels were higher in the HIP group vs. CG (p<0.0001). In HIP, linear analysis of the evaluated parameters identified direct correlations between hs-CRP- erytrocites sedimentation rate (ESR) (p=0.04), number of ART regimens and PIs exposure (p=0.007), IMT (p=0.000) and HIV-RNA (p=0.000) and also between IMT – triglycerides (p=0.004), PIs exposure (p=0.004), CD8+ (p=0.0000) and HIV-RNA (p=0.001).

## Conclusions

Young HIP have had an average value of hs-CRP equivalent with a moderate CVR; the CVR profile in young seropositive infected with HIV-1 during infancy includes elevated triglycerides, ESR, CD8+, HIV-RNA values and long time of PIs exposure. IMT in young HIP experienced to ART suggests a premature "aging" of the vessel by about two decades.

